# Linking Adult Second Language Learning and Diachronic Change: A Cautionary Note

**DOI:** 10.3389/fpsyg.2018.00480

**Published:** 2018-04-05

**Authors:** Vera Kempe, Patricia J. Brooks

**Affiliations:** ^1^School of Social and Health Sciences, Abertay University, Dundee, United Kingdom; ^2^College of Staten Island and The Graduate Center, City University of New York, Brooklyn, NY, United States

**Keywords:** linguistic niche hypothesis, first language acquisition, second language learning, inflectional morphology, case marking

Traditionally, diachronic language change has been attributed to intra-linguistic factors, which, in analogy to genetic drift, result in diversification of languages as a consequence of the social and geographical separation of linguistic communities (Lupyan and Dale, 2016). More recently, extra-linguistic factors have been implicated in language change as languages adapt to ecological niches formed by geographic, demographic, and cultural characteristics of social environments (Dale and Lupyan, 2012; Reali et al., 2018). One way of conceptualizing these extra-linguistic factors is to distinguish linguistic communities along a continuum of variation in population size, geographical spread, and amount of contact with other languages: Inward-facing, esoteric communities have small populations with shared knowledge and little language contact whereas outward-facing, exoteric communities have large populations, assembled into diverse social networks with substantial amounts of non-shared knowledge and contact with other languages (Thurston, 1987; Wray and Grace, 2007).

According to the Linguistic Niche Hypothesis (Lupyan and Dale, 2010; LNH: Dale and Lupyan, 2012), larger proportions of non-native speakers in exoteric communities promote morphological simplification of the majority language. This is thought to occur because simplifying adjustments to non-native interlocutors produced by native speakers (Little, 2011) or linguistic forms better adapted to learning constraints of adult second-language (L2) learners are adopted and transmitted to subsequent generations. Support for this hypothesis comes from qualitative (McWhorter, 2007; Trudgill, 2011) and quantitative (Szmrecsanyi and Kortmann, 2009; Lupyan and Dale, 2010; Bentz and Winter, 2013) analyses suggesting a negative correlation between the proportion of L2-learners in a linguistic community and the morphological complexity of the majority language (but see Nichols, 1992; Atkinson et al., 2016, for failures to observe this link). Below we evaluate evidence for this proposal, consider an alternative, and suggest directions for future research.

Adult language-learners differ from children in terms of prior real-world knowledge and literacy levels. Such differences allow adults to map L2s onto fully developed conceptual and linguistic representations, and may render them oblivious to aspects of morpho-syntactic structure that are not present in their L1, especially if not underpinned by awareness gained through literacy (Tarone et al., 2007). Adults and children also differ in learning mechanisms: Children rely on procedural memory whereas adults utilize declarative memory, at least in the initial stages of L2 grammar learning (Hamrick et al., 2018). Finally, relative to adults, children's cognitive limitations restrict their ability to consider contextual and referential information (Trueswell et al., 1999; Snedeker and Trueswell, 2004; Weighall, 2008). Nettle (2012) has conjectured that, as a result, children might benefit more than adults from over-specification afforded by redundant cues in complex morphological systems. Indeed, for at least one esoteric language, Choguita Rarámuri, processing benefits have been observed from redundant morphological marking in situations where meanings of constructions are difficult to recognize (Caballero and Kapatsinski, 2015); however, evidence that benefits from over-specification are amplified in children is lacking. To test how learning and processing differences between adults and children shape morphology, the LNH has operationalized morphological complexity through estimates of the amount of morphologically-marked grammatical features and bound morphemes marking those features (Lupyan and Dale, 2010; Bentz and Winter, 2013).

Yet how strong is the evidence that children's cognitive limitations support learning of complex morphology? According to the “Less-Is-More” hypothesis (Newport, 1990), limited processing capacity focuses children's attention on smaller chunks of the input, facilitating its decomposition into sublexical units, such as inflectional affixes, and the mapping of these units onto grammatical features. Adults, in contrast, tend to process larger chunks of input, which may prevent them from noticing fine-grained variation crucial for learning inflectional morphology. Evidence for adults' limited decomposition ability has mainly been obtained from studies comparing the processing of regularly inflected vs. irregular forms (e.g., English past-tense verbs, German past participles). Evidence from priming and ERP studies suggests that native speakers rapidly decompose inflected regular forms into constituent stems and affixes, whereas adult L2-learners treat both regular and irregular forms as unanalyzed wholes (Clahsen et al., 2010), presumably because their initial reliance on declarative memory taxes cognitive resources and thus constrains the complexity of what can be learned (McDonald, 2006; Hamrick et al., 2018). Morphological complexity is also thought to impose a burden on production because it commits speakers to engage in additional “thinking for speaking,” i.e., the obligatory encoding of information that may go beyond their immediate communicative intentions (Slobin, 1996, 2003). However, direct empirical support for the idea that cognitive limitations confer advantages for learning complex morphology is lacking: First, we know of no study that has directly compared children vs. adults in their tendency to decompose unfamiliar pseudo-linguistic stimuli. Second, neither connectionist models that varied memory capacity (Elman, 1993) nor experimental studies that imposed concurrent cognitive load on adult language learners (Cochran et al., 1999) yielded unequivocal and replicable evidence for superior decomposition or faster learning of morpho-syntax as a consequence of processing capacity limitations (Rohde and Plaut, 1999, 2003). There is to date no convincing evidence that cognitive limitations benefit input decomposition as an aid to morphology learning.

A related proposal attributes children's language-learning advantage to limitations in cognitive control (Thompson-Schill et al., 2009; Chrysikou et al., 2011). When exposed to artificial languages with competing variants of free morphemes distributed in unpredictable ways, children typically regularize the input by dropping less frequent variants, whereas adults tend to probability-match, i.e., to reproduce the statistical distribution of morpheme variants in the input (Hudson Kam and Newport, 2005). Such results suggest that children's inability to inhibit pre-potent responses may lead to regularization of unpredictable variation of the type encountered in pidgins. However, direct attempts to induce regularization in adults by imposing concurrent cognitive load have been unsuccessful (Perfors, 2012), suggesting that regularization is not a consequence of limitations in processing capacity and executive control, but rather a strategic response (Perfors, 2016). Additionally, while children's propensity to regularize may play an important role in creolization, it is unclear how it could facilitate morphology acquisition in natural languages, given that morphological structure has evolved to be quasi-regular and largely predictable (Kirby et al., 2015). If children were to regularize complex morphological systems, this would lead to neutralization of features and erosion of morphological contrasts—a prediction that contradicts the idea that children drive morphological complexity. Adults, on the other hand, regularize only at much higher levels of complexity, and only when variation is truly unpredictable, but not when it resembles the lexically-conditioned morphological variation of natural languages (Hudson Kam and Newport, 2009). This and other evidence that adults are quite capable of learning complex morphological systems, adopt similar learning strategies as children, and may even often outperform children in controlled experimental studies (Braine et al., 1990; Brooks et al., 1993; Wonnacott et al., 2008; Wonnacott, 2011) is difficult to reconcile with the idea that non-native speakers of a language are responsible for the erosion of its morphological complexity. Moreover, for simpler morphological patterns to become established in a language, the changes must be adopted by the next generation of L1-speakers. Although the children of non-native speakers may regularize their parents' unpredictable input, this process may not yield a less complex system, as documented in case studies of children acquiring sign language (Singleton and Newport, 2004).

Other accounts have emphasized that the morphological features of languages used by esoteric communities are idiosyncratic, low in compositionality, and replete with irregularities and formulaic expressions (Wray and Grace, 2007). Such systems arise because members of esoteric communities share a great extent of knowledge, which enables them to use contextual cues to discern utterance meanings and leave the linguistic expressions themselves more ambiguous. While this view would be compatible with the general idea of language adapting to a sociocultural niche, it is at odds with the idea that redundant marking of grammatical features by bound morphemes is the relevant characteristic of esoteric communication (Lupyan and Dale, 2010). Instead, it leads to an alternative prediction: that morphological systems acquired predominantly by children should be more idiosyncratic and less transparent than the regular, transparent, and compositional morphological systems preferred by adult L2-learners. This alternative aligns with evidence of children's propensity to learn from larger, unanalyzed chunks (Peters, 1983; Pine and Lieven, 1993)—a proposal contradicting Newport's (1990) version of the “Less-Is-More” hypothesis. Indeed, recent evidence (Arnon and Christiansen, 2017; Arnon et al., 2017) suggests that due to limited processing capacity and lack of conceptual knowledge, children may under-segment the input and form representations of multi-word utterances along with their constituent components. Such concurrent representations enable children to harness predictive information inherent in the constituent components, which benefits learning of adjacent dependencies such as Spanish determiner-noun gender agreement (Arnon and Ramscar, 2012) or Chinese classifier-noun associations (Paul and Grüter, 2016). Even if adults form representations of multi-word utterances through chunking, their already existing conceptual knowledge may lead them to miss out on the predictive information from free morphemes contained in these utterances, focussing instead on the mapping of novel L2 content words onto existing concepts. However, while children's learning from larger, only partially decomposed multi-word utterances can explain acquisition of grammatical features marked by predictive free morphemes (e.g., determiners, prepositions), it does not explain the acquisition of bound morphemes, which are at the heart of the LNH.

A possible way to reconcile the different conceptualizations of how esoteric vs. exoteric communication affects language change is to acknowledge that transparency and complexity of morphological systems are orthogonal dimensions that jointly affect learnability, irrespective of whether instantiated by bound or free morphemes. Consider the following example: German nominal morphology comprises free (determiners) and bound (suffixes) morphemes marking number (singular, plural), gender (masculine, feminine, neuter) and case (nominative, genitive, dative, accusative), yet a considerable degree of neutralization and inflectional syncretism in its declension paradigm renders case markers fairly non-transparent and uninformative. In contrast, Russian nominal inflections are considerably more complex, with suffixes varying according to number (singular, plural), gender (masculine, feminine, neuter; with further inflectional variation for several nominal subclasses), and case (nominative, genitive, dative, accusative, instrumental, locative), yet the degree of neutralization and inflectional syncretism is substantially lower, rendering case markers more transparent and informative. If complexity is the main obstacle for adults learning nominal morphology, then L2-learners should exhibit greater difficulty with Russian than with German. If, however, lack of transparency poses the challenge, then German should be more difficult for L2-learners. Comprehension tasks comparing adult learners of Russian and German with comparable levels of L2-proficiency revealed that L2-learners of Russian processed case markers much more efficiently than L2-learners of German (Kempe and MacWhinney, 1998). When potential confounds between different L2s were controlled by manipulating features of morphological systems within languages, native English speakers who learned Russian case inflections for transparently gender-marked nouns progressed much faster than those who learned inflections for non-transparently gender-marked nouns, even though the two subsystems were of comparable complexity (Kempe and Brooks, 2008). These findings align with evidence that learners are biased toward morphological systems that maximize communicative efficiency (Fedzechkina et al., 2012) and suggest that conceptualizations of morphological complexity need to consider the informativeness of morphemes as cues to underlying syntactic and semantic structure (Bates and MacWhinney, 1989)—an approach compatible with connectionist (Kempe and MacWhinney, 1998, 1999; Mirković et al., 2011) and information-theoretical approaches to learning and processing of inflectional morphology (Milin et al., 2009).

To provide more stringent tests of the role of child vs. adult learners as drivers of morphological change, a cognitively-grounded typology of informativeness—obtained through quantitative approaches—is needed, for example, using connectionist or deep-learning algorithms that estimate strength of association between morphological markers and thematic roles from morphologically-tagged language corpora, or inferential algorithms that operate on probability distributions of markers over thematic roles in analogy to what has been suggested for semantic typology (Kemp et al., 2018). Such estimates should be integrated with findings from cross-linguistic studies of how adults and children learn and process different morphological systems to complement existing models of learner biases in terms of exposure (Bentz and Berdicevskis, 2016) or preference for regularization (Cuskley et al., 2017), while taking into account more subtle differences in children's cognitive and pragmatic capacities. We expect especially strong insights to be gained from amplification of adult vs. child biases during transmission of language in iterated learning studies. Initial forays into this line of inquiry indicate that compositional morpho-syntax emerges more readily when systems are learned and transmitted by adults than by children (Flaherty and Kirby, 2008; Raviv and Arnon, 2016), suggesting that at present questions about how languages adapt to different learnability constraints imposed by children and adults are far from settled.

## Author contributions

All authors listed have made a substantial, direct, and intellectual contribution to the work, and approved it for publication. The authors developed the ideas jointly and collaborated in writing the article.

### Conflict of interest statement

The authors declare that the research was conducted in the absence of any commercial or financial relationships that could be construed as a potential conflict of interest.
